# A Longitudinal Exploratory Case-Series Comparing Morphological Trajectories in Four Early-Implanted Greek-Cypriot Children and Eight Typically Hearing Peers

**DOI:** 10.3390/brainsci16090939

**Published:** 2026-09-01

**Authors:** Elena Yiangou, Marianna Christodoulou Devledian, George Spanoudis

**Affiliations:** 1 Department of Health Sciences, European University Cyprus, Nicosia 2404, Cyprus; mari.christodoulou@euc.ac.cy; 2 Department of Psychology, University of Cyprus, Nicosia 1678, Cyprus; spanoud@ucy.ac.cy

**Keywords:** cochlear implants, morphological development, Cypriot Greek, MLUm, definite article

## Abstract

**Highlights:**

**What are the main findings?**

- Seemingly asynchronous growth spurts between cochlear-implanted children and typically hearing children at certain time points reflecting the younger hearing age of the children with cochlear implants;
- A delay in access to auditory stimuli impedes the acquisition of the definite article at first. Once auditory access is granted children portray their growth spurts at a slightly later time compared to typically hearing children due to early implantation;
- MLUm and TTR growth seems to be aligned between the two groups.

**What are the implications of the main findings?**

- Adding to the existing literature on the benefits of early implantation;
- Provides preliminary results on deficits that may appear in developing cochlear-implanted children that may be within their morphological developmental path;
- Persistence of these deficits or deficits in certain cases of the definite article, e.g., nominative case, may indicate an emerging language deficit.

**Abstract:**

**Objectives:** This paper explores whether deaf Greek-Cypriot (GC) children with cochlear implants (CIs) begin using the various forms of the definite article (DA) of the Greek language similarly to their typically hearing peers in terms of order of appearance and age. Furthermore, it explores if their MLUm growth correlates with that of typically developing peers. **Methods:** This is a longitudinal study with quantitative and qualitative analyses. The sample comprises four prelingually deaf GC children with CIs and eight typically hearing (TH) children with no developmental delays. Age and hearing status (CIs vs. TH) were used to explain variability in results. The effect of random effects was also examined. Presence of random effects was examined using Mixed Linear Models. Growth trajectories were plotted for in-depth result description and interpretation. **Results:** Both groups demonstrated a significant linear increase in their accurate usage of definite articles with age, whereas hearing status showed no statistically significant impact on any outcome measure. Individual variance among children accounted for approximately 33% to 50% of the variance in correct article production. Additionally, Mean Length of Utterance in morphemes (MLUm) values between the two groups remained correlated across all age levels. **Discussion:** While random individual effects influence the rate of correct production, early morphosyntactic trajectories for CI children seem to be similar to those of typically developing controls. Due to the small sample size, studies with bigger samples are needed to replicate, confirm and validate further the results of the current study.

## 1. Introduction

Over the past few decades, spoken language has emerged as both a highly effective and preferred method for deaf children with cochlear implants raised in hearing families [1]. Clear access to auditory stimuli has been found to aid speech perception and promote stronger spoken language skills [2]. Constant exposure to auditory stimuli [3] and high correlation between spectral discrimination of auditory stimuli and speech perception [4] have been shown to aid in better perception of vowels and consonants and development of executive functions. Examples include processing of auditory stimuli and sustained attention in environments with poor acoustic properties [5], and the development of pragmatics [6,7]. Exposure to auditory stimuli in profoundly deaf children is achieved using cochlear implants by laying the foundation for language development [8,9,10].

### 1.1. Sensitive Periods, Critical Periods and Language Development

Spoken language acquisition depends entirely on auditory access—a critical foundation that cochlear implants restore for deaf children. The extensive literature supports acquisition of spoken language post implantation [11,12,13,14] while a growing number of studies support early implantation [15,16,17,18,19]. Eary implantation has been associated with a sharper increase in receptive language skills [20] and faster vocabulary growth, lending support to the existence of critical periods [21,22]. A critical period is a time-limited window of neuroplasticity essential for the optimal development of specialized functions, such as audition and language. Deprivation of auditory input beyond this critical period compels the brain to reorganize, shifting its neural pathways to process information visually [23,24]. A reason why early implantation is so effective in facilitating spoken language is possibly the fact that the auditory cortex does not degrade structurally in congenitally deaf people. According to the literature review by Hribar et al. (2020), substantial evidence indicates that cross-modal neuroplasticity enables the auditory cortex to retain its structural integrity through functional reorganization driven by alternative sensory modalities [25]. One example given by Benetti et al. (2017) is the auditory cortex being activated during recognition of different faces as it would in typically hearing people during recognition of different voices [26]. These findings have led to a change in FDA regulations where congenitally deaf children in the US can be implanted as young as 9 months old [27]. Even though regulations in Cyprus abide by FDA regulations, the available sample at the time of this study were children implanted at the age of 12 to 15 months.

While pediatric cochlear implantation outcomes are extensively researched in English, a major empirical gap persists for Greek. This study attempts to address this limitation by examining morphological development in deaf children using cochlear implants who speak the GC dialect. Morphological competence is paramount due to its complexity and structural necessity within the language, a position reinforced in Section 1.1 and Section 1.2 through examination of Greek morphology and the functional role of the definite article.

### 1.2. Overview of Greek Language Morphology

Greek inflection resembles that of languages like German and Portuguese, requiring frequent, obligatory grammatical morphemes to convey precise meaning and license semantic inferences, in contrast to the English language which comprises a much simpler inflectional system [28]. Children learn to add morphemes to the main word stem through the process of inflection. Efficacy in the use of morphemes is of utmost importance in language acquisition since it has been found to predict acquisition of literacy aspects, such as spelling. Successful use and morpheme comprehension are mirrored in increased successful spelling in school-aged children. This is particularly evident in languages where the word spelling matches the morphological rules it abides by [29]. In Greek, bound morphemes often fuse multiple grammatical features; for instance, the suffix *-ος* in *γάτος* /γatos/ (‘cat’) simultaneously encodes masculine gender, singular number, and nominative case. Similarly, in the verb *γράφω* /γrafo/, the lexical stem *γραφ-* combines with the grammatical suffix *-ω* (first-person singular present tense) [30].

A major typographic difference highlights the vital role of morphology in Greek compared to English. In English, structural syntax determines thematic roles: in a standard transitive sentence, the noun preceding the verb is designated as the subject. Conversely, Greek relies on morphological case rather than word order to assign grammatical functions, where the nominative case identifies the subject [28]. In the absence of case marking, semantic relations cannot be parsed, causing complete grammatical ambiguity for the listener. Furthermore, each noun requires an accompanying definite article, with obligatory number, case, and gender agreement [28,29].

### 1.3. Definite Article Function and Use in Greek and Other Highly Inflected Languages

Definite article use seems to begin around the age of 1 year 7 months. Marinis looked at the acquisition of the definite article in MG by adopting Nominal Mapping Parameter posed by Ghierchia on feature specification of nouns, “based on the idea that the syntax-semantics mapping in the nominal system is not cross-linguistically uniform” [31,32,33,34] (p. 1). He used two corpuses. The data includes the Ursula corpus, focusing on definite article acquisition in four children aged 1 year; 9 months to 2 years; 9 months, and the Christofidou corpus, which observed one child’s language growth from 1 year; 7 months to 2 years; 7 months [33]. These results align with Szagun et al., (2007) who found that German-speaking children begin using gender-marked definite articles as early as 1 year, 5 of age [35].

A possible explanation for the early use of the definite article is the frequent adult input they receive in the morphologically complex language environments they are raised in [36]. This is supported further in a study by Lew-Williams and Fernald where children seemed to have improved accuracy and faster response times in locating the object within a noun phrase when the definite article preceding the noun carried gender-marking information [37]. It is, therefore, important to know the age at which children acquiring Greek language begin using the definite article, as it may be a potential marker for receptive and expressive language development in highly inflected languages.

Acoustically, the definite article is not salient and is similar when changing from nominative to accusative, e.g., *ο* to *το*, */ο/* to */tο/*, a common feature shared with the German article system [38]. Therefore, cochlear-implanted Greek children are expected to have difficulties in its acquisition, unless growing up in a rich morphologically linguistic environment offsets this expected difficulty. Furthermore, it may be reasonably inferred that correct and early use of the definite article in implanted children may be an indicator of a probably unremarkable receptive and expressive language development. This should presumably apply to the GC dialect as well since they share the same grammatical rules and structure.

### 1.4. Greek-Cypriot Dialect

The dialect of the Greek language spoken in Cyprus is Greek Cypriot. In schools, both in Greece and Cyprus, the standard language used for instruction is Modern Greek (MG). GC and MG do not differ significantly. One of the main differences is in the syntax of GC dialect where double clitics are used in sentences. For example, in MG, the sentence ‘He didn’t give me the letters’ would be *Δεν μου έδωσε **τα** γράμματα /ðen mu edose **ta** γrammata/.* In the aforementioned sentence the only clitic used is the definite article *τα /ta/*. In GC the sentence becomes *Εν μου **τα** έδωσε **τα** γράμματα /En mu **ta** edose **ta** γrammata/,* demonstrating double use of the clitic [39]. Furthermore, the initial sound for the word */ðen/* drops and the word becomes */en/* in GC, indicating a difference in phonology.

Studies on the GC dialect, amongst others, published to the present date examined object and action naming in Greek-Cypriot children [40], subject relatives (SRs) and object relatives (ORs) acquisition [41], the efficacy of MG SLI diagnostic tools adapted to the GC dialect, used to diagnose SLI in GC-speaking children [42], pinpointing possible factors that may play a role in language development of pre-schoolers speaking the GC dialect and their linguistic characteristics [43], and how use of colorful semantics affects the morphosyntactic and semantic development in GC children with autism [44]. Presently, to the best of our knowledge, there are no published studies examining the definite article acquisition in typically hearing children or children with CIs speaking the GC dialect.

### 1.5. Mean Length of Utterance in Morphemes

One way to assess language development is to evaluate the acquisition and use of vocabulary. An additional way for a highly inflected language like MG is the acquisition of morphemes. Mean Length of Utterance in morphemes (MLUm) is a widely used general language development index, which measures morpheme use and overall grammatical development [45,46,47]. Substantial research supports the selection of this metric, highlighting its methodological advantages as a naturalistic assessment that minimizes dialectal bias [46]. Brown argued that children’s MLU level better mirrors their language abilities rather than their chronological age. Furthermore, children’s MLU changes as they get older in a rather foreseeable manner, rendering it a useful tool for placing children in specific age brackets [45].

MLU was designed for English, creating a caveat when applied to highly inflected languages with rich morphology. One of Brown’s [45] guidelines states that only morphemes signifying productive use should be counted. In highly inflected languages however, this guideline is nearly impossible to apply due to the high scarcity of studies investigating the morphological development of children born in languages with rich inflection. As a result, use of MLU in words is used more frequently in such languages to bypass this limitation (Mean Length of Utterance in words; MLUw). Even so, highly inflected languages like Icelandic have adapted Brown’s rules to fit their specific inflectional systems, allowing them to measure MLU in morphemes. Research in Greek-speaking children with CI and TH children shows a correlation between MLU scores in sonority and prosodic word categories of expressive and receptive vocabulary for children aged 21–71 months [48]. Further, Type/Token Ratio (TTR) is a key measure of lexical diversity within a text or person’s speech assessed by comparing unique words to total words. A psychometric study by Fergadiotis et al. on the effectiveness of diverse lexical diversity measures revealed that a specific Type–Token ratio is closely linked to the lexical diversity construct, indicating its utility as a valid proxy [48,49].

The scarce use of MLU applies to studies investigating morphological development in children speaking the GC dialect. Voniati (2016) analyzed language samples from 36 monolingual Greek-Cypriot (GC) children aged 36 to 48 months using Mean Length of Utterance in words (MLUw) rather than morphemes (MLUm), citing established challenges in applying MLUm to highly inflected languages [50]. Consequently, the present study represents the first effort to apply MLUm to the GC dialect. Lastly, MLU has also been applied to track language growth in deaf children who use cochlear implants [51].

Research on Greek children with CIs are limited. These studies, amongst others, have focused mainly on vocabulary acquisition [52], training and knowledge of speech-language therapists who work with young CI recipients [53], short-term memory in children with cochlear implants [54] and speech production of hearing-impaired children [55].

The scarcity of research on GC deaf children with CIs, paired with the definite article’s critical role as a marker for morphological development, underscores the significance of the current study. Despite the limited literature on morphosyntactic acquisition in this population, existing data suggests that GC-speaking children with CIs should demonstrate early use of the definite article, provided their linguistic development mirrors that of typically developing peers.

Thus, we formulated the following research questions:

(1) Do all participants, irrespective of hearing state at birth, exhibit an overall increase in MLUm scores and correct occurrences of the definite article in all its forms and does participants’ age affect the direction in which their MLUm scores and number of correct definite article (DA) occurrences move?

(2) Is there a difference between children with and without CIs in the early pattern of correct productions of the definite article in the nine forms examined?

(3) Is there a difference between children with and without CIs in their morphosyntactic level as measured using MLUm, MLUw and TTR?

## 2. Materials and Methods

### 2.1. Participants

The sample comprised four congenitally deaf GC children with CIs (two boys and two girls) of ages 2 years to–2 years; 3 months, and eight TH peers. A fifth GC child with bimodal amplification was part of the initial sample but withdrew from the study within the first year of data collection due to her inability to complete the tasks. All children resided in the three main cities in Cyprus. Children with CIs and TH children were matched on gender, chronological age, city of residence and their status of being born in monolingual families of GC. Although small in absolute numbers, the sample constitutes the entire available population under investigation at the time the study commenced. A larger number of TH children (THn = 8) were recruited to avoid having less than four children participating in case of attrition. Table 1 depicts the participant demographics of the CI group.

### 2.2. Inclusion Criteria

Eligible candidates for the CI group were required to:

(1) Be monolingual speakers and come from monolingual families of the Greek-Cypriot dialect;
(2) Have received one of the two or both cochlear implants by the age of 12 to 15 months;
(3) Have not had any other comorbid diagnoses of other syndromes or disabilities other than deafness;
(4) Be a chronological age of 2 years old at the commencement of the study.

Eligible candidates for the TH group were required to:

(1) Have no diagnoses of any disabilities or syndromes;
(2) Be 2 years old at the commencement of the study;
(3) Demonstrate typical language development per teacher and parent report, and the use of the Communicative Development Inventory (the adapted version of the McCarthur Communicative Development Inventory to the Greek-Cypriot dialect [56,57]. Children had to have 50 words on their expressive vocabulary as per parent report, indicating normal levels of development for their age. Due to lack of standardized language assessments on Greek-Cypriot children, CDI had to be used as a baseline measure;
(4) Demonstrate typical auditory awareness and response skills as measured by the LittlEars© Questionnaire [58];
(5) Demonstrate typical levels of hearing at the commencement of the study;
(6) Not have had >2 occurrences of otitis media.

Children with CIs were recruited from Nicosia General Hospital and Audiological Center of Cyprus. Interested participants contacted the primary investigator and signed consent after reviewing all applicable personal data regulations. TH children were recruited from three different kindergartens in the three main cities in Cyprus. Kindergarten principals were briefed on the specifics of the study and signed the consent form. They then contacted families whose children met the inclusion criteria.

### 2.3. Measures

#### MLUm

Mean Length of Utterance in morphemes was used to measure participants’ Mean Length of Utterance in morphemes. One hundred utterances were used for MLUm calculation. We analyzed language samples using Brown’s [43] general criteria alongside newly established rules tailored specifically to Greek’s rich inflectional system. For example, in English, pronoun *he* counts as one morpheme. The same pronoun in Greek, *αυτός /aft*ɔ:*s/,* was modified to count as 4 morphemes because the ending signifies four different grammatical aspects: noun + person + number + case. Consequently, it can be anticipated that MLUm values for GC-speaking children will exceed those of English-speaking children across Brown’s developmental stages. MLUm scores between English and GC dialect are therefore neither directly comparable nor expected to align. For this reason, Brown’s stages were not utilized as a normative benchmark for typical language development in the present study. Please see Appendix A for a full list of the modifications of the coding rules.

### 2.4. Definite Article Use

The definite article was elicited using a protocol consisting of two elicitation scenarios, developed for the execution of the Structured Session (SS) of this study. The nature and proceedings of the SS are described in the Procedures Section below. Each form of the definite article, e.g., masculine, in the nominative case in singular, had a total of two obligatory contexts with the exception of the neutral gender singular in the nominative case which only had one.

### 2.5. Procedures

The study consisted of two main sessions for each participant: Language Sampling Session (LS) and Structured Session (SS).

#### 2.5.1. Language Sampling Session

A 30 min language sample was collected from each participant in six-month intervals, from the age of 2 years until the age of 4 years; 6 months. The child was presented with three bags every 10 min in a consecutive order. The child was allowed to explore and play with the contents of each bag for 10 min. Allocating equal time to each bag ensured that the procedure was as standardized as possible across all participants [59].

The first bag contained the book, “Monkey Puzzle”. The prompt, *"Το πιθηκάκι είναι λυπημένο επειδή έχασε τη μαμά του και η πεταλούδα θα το βοηθήσει να τη βρει"* (The monkey is sad because it lost its mommy and the butterfly is going to help the monkey find her) was given at the beginning of the session to instigate discussion and spontaneous commenting. At times when the child remained silent, prompts such as, *"Τι άλλο βλέπεις;"* (What else do you see?) or *"Τι νομίζεις έγινε μετά;"* (What do you think happened next?) were given to encourage the child to comment some more on each page.

The second bag contained kitchen utensils and food items. Prompts such as *"Πεινάω! Νομίζω θα φτιάξω κοτόπουλο με πατάτες"* (I am hungry, I think I will make chicken with potatoes), or *"Πεινάω! Τι νομίζεις θα ήταν καλό να μαγειρέψουμε;"* (I am hungry, what do you think we should cook?) were given to children who did not initiate play.

The third bag contained farm animals and a tractor. Prompts such as, *"Έλα να πάρουμε τα ζώα βόλτα με το τρακτέρ"* (Let’s take the animals for a drive with the tractor) or *"Για να δούμε, ποια ζώα θέλουν να πάνε βόλτα;"* (Let’s see, which animals want to go for a drive?) were given to children who did not initiate play.

All Language Sampling Sessions were recorded at the children’s preschool, except for one participant who was unable to drive to the nearest preschool and so language sampling collection occurred at her house following the same administration procedure. Before language samples were collected, the investigator spent individual time building rapport with each participant to ensure they were comfortable remaining in the testing room alone. Although involvement of a parent or teacher’s assistant was initially considered, this option was ultimately ruled out to prevent introducing additional variance into the data collection environment.

#### 2.5.2. Structured Session

##### Part One

“Monkey Puzzle” was used again to elicit the definite article in all genders, cases and numbers by creating obligatory use contexts through commenting based on the book’s pictures. Commenting was guided through the use of a script consisting of 19 items. Eight of these items used incomplete statements for DA elicitation. Each sentence frame required participants to supply a missing noun preceded by its appropriate definite article. The correct inflectional form of the article—specifically its gender, number, and case—was dictated by the syntactic structure of the prompt and the linguistic properties of the target noun (e.g., *"Πάνω στο δέντρο είναι τυλιγμένο το φίδι"* (*The snake* is wrapped around the tree)). The use of the adverb ‘on’ */pa:nə/* (πάνω) at the beginning of the sentence defines the case of the definite article, i.e., nominative. The number and gender of the definite article are defined by the noun itself, in this case, ‘το φίδι’ */τə fi:ði:/* refers to the snake, which is, i.e., neutral singular. The rest of the items (11) were analogies such as, *"Δεν φοβόμαστε τις πεταλούδες αλλά φοβόμαστε τους κροκόδειλους"* (We are not scared of (the) butterflies, but we are scared of (the) crocodiles). Syntactically the definite article ‘the’ would not be appropriate before the word ‘crocodiles’ in the English language. However, the use of the definite article is obligatory in GC. In the aforementioned example, the connective ‘but’ *αλλά /alla/* used connects two sentences consisting of subject–verb–object: The word *“εμείς*" (we) is in parentheses because it is not actually said by the examiner. It is mirrored in the ending of the verb "*φοβό- μαστε*". It is added in the breakdown of the sentence to represent the subject (*-δεν φοβόμαστε-(τις) πεταλούδες”* (we are–not scared of-butterflies) and "(εμείς)*-φοβόμαστε-(τους) κροκόδειλους" (we are-scared of-crocodiles)). The syntactic structure of the first sentence creates an obligatory context for the use of the definite article in the accusative case, i.e., *"τις" /ti:s/*. The gender and the number are defined by the noun used, i.e., butterflies (female, plural). The second sentence must have the same syntactical structure based on the semantics of the complex sentence formed; therefore, the definite article and the proceeding noun-ending required to fill in the sentence have to be in the accusative case too, *"τους" /tʊs/* (male plural). The gender of the definite article changes to match the noun gender change. The number remains the same, i.e., plural, since both nouns are in plural. Initially, an example was given to each child prior to administering test items to ensure they understood what was expected of them. Two additional examples were given if a child did not understand the first example.

The carrier phrase *"Μετά η πεταλούδα έδειξε στο πιθηκάκι έναν/μία/ένα _____"* (Then the butterfly showed the monkey a/an ______) was given for each new animal presented in the book; for example, "*Μετά η πεταλούδα έδειξε στο πιθηκάκι έναν ελέφαντα*" (Then the butterfly showed the monkey an elephant), to create an obligatory context for DA use by establishing common knowledge of the referent, i.e., the elephant. By introducing the elephant to the child, it ceases to be *an* unknown elephant and becomes *the* elephant, i.e., the subject at hand. Consequently, when the child was given the statement "*Το πιθηκάκι κοιτάζει* __________" (The monkey is looking at ____) and was asked to fill-in the blank, i.e., "*τον ελέφαντα*" */tən elef˄d˄ /* ‘the elephant’, the use of the definite article "*τον*" */tən/* became obligatory [60].

The statement was given once. If the child began talking during item administration, the investigator stopped, waited for the child to finish, and then repeated the item once the child was attentive again.

##### Part Two

During this part, a scripted play scenario with a Playmobil set was administered. The child was asked to help the farmer feed his animals (two dogs, two birds, one turtle, two butterflies). Establishing a common reference was necessary for the development of elicitation items for this part as well. The child was presented with the toy animal whose name and its accompanying definite article were the desired response. Upon presenting it to the child, the incomplete statement was given, and the child was asked to provide the missing target noun and definite article. For example, the examiner would hold up a dog and say, *"Πρώτα θα ταΐσουμε _____"* (First we will feed ______) and the child had to say *"τον σκύλο" /tən ski:llə/* (the dog). The concept behind the obligatory context of the use of the definite article in this part was the fact that young children first begin using the definite article instead of the indefinite article because they do not understand its meaning [60]. Upon completing each sentence, the child received the toy to play with; this process was repeated systematically until all stimulus items were presented. Similar to Part One, the script for Part Two utilized a combination of cloze (fill-in-the-blank) items and analogies, following the same methodological rationale.

### 2.6. Equipment

All sessions were recorded and videotaped for transcription purposes using the Tascam DR100MKII portable recorder (TASCAM/TEAC Corp., Tokyo, Japan), the Sony ECM88B microphone (Sony Corp., Tokyo, Japan), and the Zoom Q3HD portable camera (Zoom Corp., Tokyo, Japan).

### 2.7. Transcription

All language samples were transcribed by a trained research assistant. All samples were checked by the main investigator to ensure accuracy in transcription by reading the transcription while listening to the recording. Word Error Rate was calculated. Error percentage ranged from 5 to 7% of the total number of words used in the samples. These were corrected by the main investigator before commencing coding.

### 2.8. Coding

All language samples were coded by the main investigator. Due to time constraints and lack of manpower, samples could not be coded by a second rater. Instead, the coordinator of the project reviewed the coding of 20% of the samples and no major discrepancies were noted. Furthermore, during coding, the main investigator consulted with the coordinator whenever a word could be coded in two different ways to eliminate, to the best of our abilities, the occurrence of any errors.

### 2.9. Statistical Analyses

We employed a series of Mixed Linear Models (MLM) to accommodate our longitudinal data structure, which featured unequal sample sizes across our two-group design. This approach allowed us to evaluate overall population trends via fixed effects while simultaneously modeling for participant-specific variability over time through random effects. To account for our small overall sample size (n = 12) and prevent inflation of Type I error rates, we used the pbkrtest package [61] to apply the Kenward–Roger approximation for adjusting the standard errors and denominator degrees of freedom for the fixed effects [62].

## 3. Results

Given the very small number of children with cochlear implants (N = 4), the analyses reported below are exploratory and descriptive rather than confirmatory, and should be interpreted as preliminary, hypothesis-generating observations rather than robust or generalizable estimates of group differences [63]. Under this approach, all participants were first analyzed as a single cohort—regardless of hearing status at birth—across MLUm, MLUw, and TTR and all nine individual forms of the Greek definite article shown in Table 2, before hearing status was introduced as a predictor.

### 3.1. Models Used

To address the longitudinal trajectories of the 12 linguistic measures across the six testing intervals, a series of linear mixed-effects models were fitted using Restricted Maximum Likelihood (REML) estimation. Specifically, a series of three progressively constrained hierarchical linear models was constructed. This analytical framework was systematically positioned to isolate overall data variability before quantifying the exact proportions of variance accounted for by chronological time, individual hearing status, and their interaction. Hypothesis testing for the random effect structures (evaluating participant-specific variation in slopes over time) was performed via Likelihood Ratio Tests (LRTs). Hypothesis testing for the fixed effects (time, hearing condition, and their interaction) was conducted using Kenward–Roger adjusted *F*-tests. This approximation method adjusted the denominator degrees of freedom to provide robust, conservative statistical inference appropriate for the small sample size and unbalanced group structure.

#### 3.1.1. Unconditional Means Model

The baseline unconditional means model divided the total variance for each dependent variable—specifically MLUm and the nine distinct forms of the Greek definite article (SSMNT, SSFNT, SSNtNT, SSMAT, SSFAT, SSNtAT, SSMGT, SSFGT, SSNtGT)—without predicting factors. This initial step determined the baseline presence of within-subject (random effects) and between-subject (fixed effects) variations. Calculating the Intraclass Correlation Coefficient (ICC) revealed the proportion of variance attributable to nested data levels. The fixed-effect intercept reached statistical significance across all dependent variables (*p* < 0.0001), confirming substantial baseline differences between participants. Specifically, for six out of the 12 outcome measures (SSMAT, SSMNT, SSFNT, SSNtNT, SSNtGT, and MLUm), within-subject variance accounted for 3–26% of the fluctuation, while the remaining 74% of the variance was driven by differences between subjects.

#### 3.1.2. Unconditional Growth Model

In the second step, chronological age—operationalized in six-month increments—was introduced into the unconditional growth model. This allowed for the assessment of longitudinal trajectories to determine whether rates of change across time varied within and/or between participants, independent of hearing status. The fixed effect of age was highly significant across all dependent variables (*p* < 0.0001). Specifically, advanced age was strongly associated with a higher frequency of correct definite article productions across all distinct forms, alongside a parallel increase in MLUm values (see Table 3). The relationship between age maturity and all language outcome measures was strictly linear.

#### 3.1.3. Conditional Growth Model

In the final model, hearing status at birth (CITH) was introduced as a predictor to assess whether—and to what extent—it accounted for between-person or within-person variance. The 12 dependent variables exhibited two distinct statistical profiles: a primary cluster demonstrating significant time + hearing condition interaction effects, and a secondary cluster characterized by parallel developmental trajectories across groups. A comprehensive summary of all computed *p*-values is provided in Table 4.

### 3.2. Group Growth Trajectories

For the majority of the continuous outcome variables—specifically SSMNT, SSFNT, SSNtNT, SSMAT, SSFAT, SSNtAT, SSMGT, SSFGT, and SSNtGT—the Kenward–Roger adjusted tests revealed statistically significant fixed interaction effects between time and hearing condition (*p* < 0.05). These significant interactions indicate that the rate of change over the six measurement waves systematically differed depending on the child’s hearing group. Because the presence of a significant interaction establishes that the effect of time is conditional upon group membership, the main effects for these nine variables are not interpreted in isolation.

The Likelihood Ratio Tests evaluating individual-level random slopes for time revealed distinct patterns of individual variation within this interactive cluster: for the measures SSMNT (*p* = 0.00003), SSFNT (*p* = 0.00042), SSFGT (*p* = 0.00010), and SSNtGT (*p* = 0.00049), the random slopes significantly improved model fit. This indicates that independent of group placement, individual children demonstrated unique, idiosyncratic baseline scores (random intercepts) as well as significantly different individual rates of progression over time (random slopes). Conversely, for SSNtNT (*p* = 0.09624), SSMAT (*p* = 0.87263), SSFAT (*p* = 0.28569), SSNtAT (*p* = 0.12519), and SSMGT (*p* = 0.25599), the random slope parameters were statistically non-significant. While children varied significantly in their initial baseline skills at session one, their individual growth trajectories over time progressed in a highly uniform and parallel manner around their respective group’s average slope.

A secondary profile was observed for Mean Length of Utterance in morphemes (MLUm), Mean Length of Utterance in words (MLUw), and Type–Token Ratio (TTR). For these three dependent variables, the fixed interaction effect between time and hearing condition was non-significant (*p* = 0.120, *p* = 0.142, and *p* = 0.119, respectively). This establishes that the rates of development across the six testing windows were statistically parallel between the typically hearing and cochlear-implanted groups.

Given the absence of conditional interaction effects, the fixed main effects were directly evaluated using the additive model structures. A highly profound, significant main effect of time was observed across all three variables (*p* < 0.0001), indicating strong, systematic developmental progression across the overall sample as the study progressed. The Kenward–Roger tests revealed a significant fixed main effect of group membership across all three parallel measures. This indicates a consistent baseline gap between the two cohorts across the entirety of the longitudinal timeframe. This systematic group difference was highly significant for TTR (*p* = 0.003), followed by MLUm (*p* = 0.031) and MLUw (*p* = 0.049). The LRTs for MLUm (*p* < 0.0001), MLUw (*p* < 0.0001), and TTR (*p* = 0.005) confirmed the necessity of retaining random slopes for time. This proves that individual children still maintained highly unique baseline positions and individual growth velocities, even though the average trajectories of the two groups remained structurally parallel.

**Figure 1:**
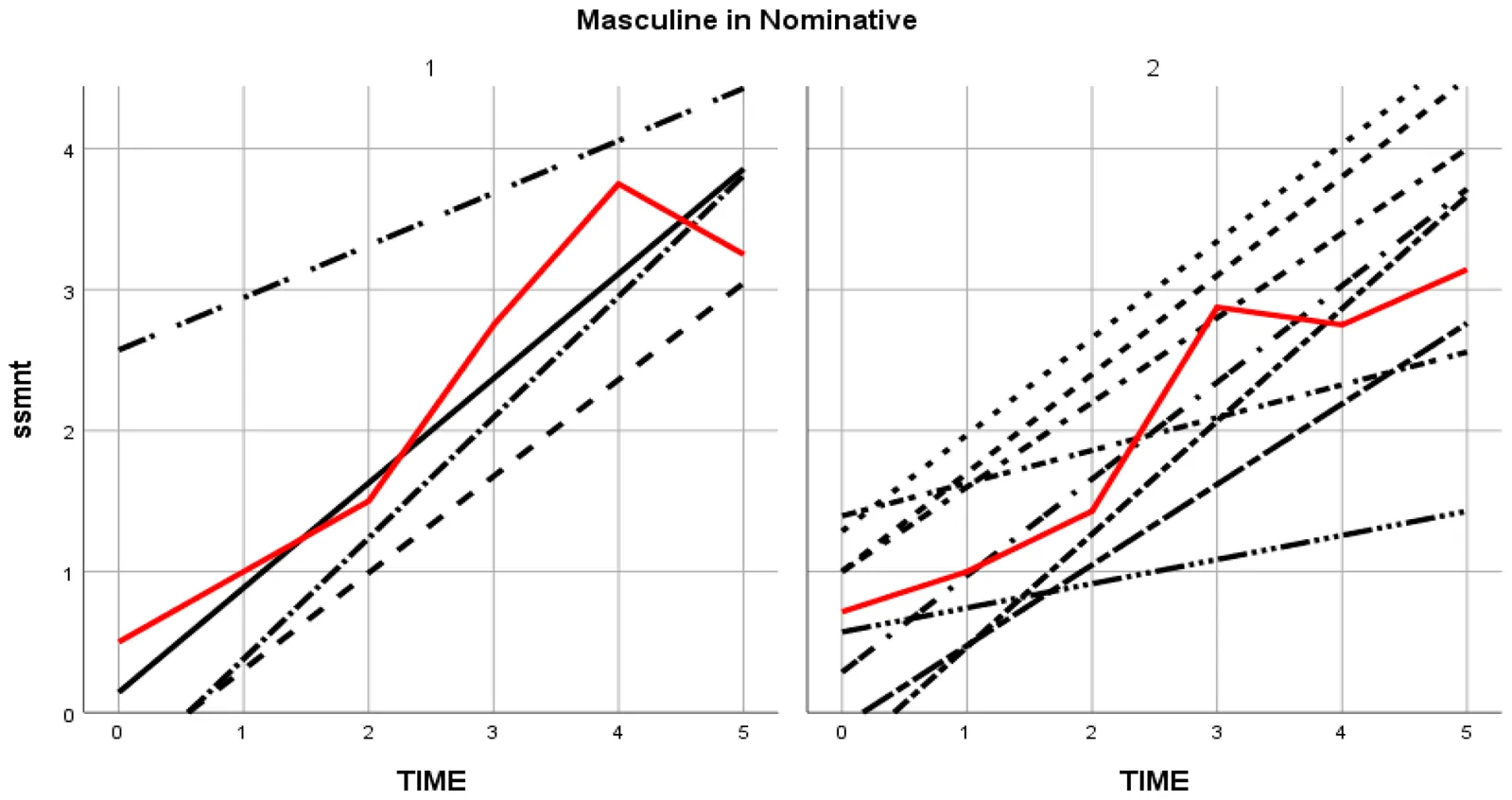
SSMNT group growth trajectories showing each group’s mean growth trajectory for the acquisition of the definite article used for the masculine gender in the nominative case. Graph 1 = CI group, Graph 2 = TH group.

**Figure 2:**
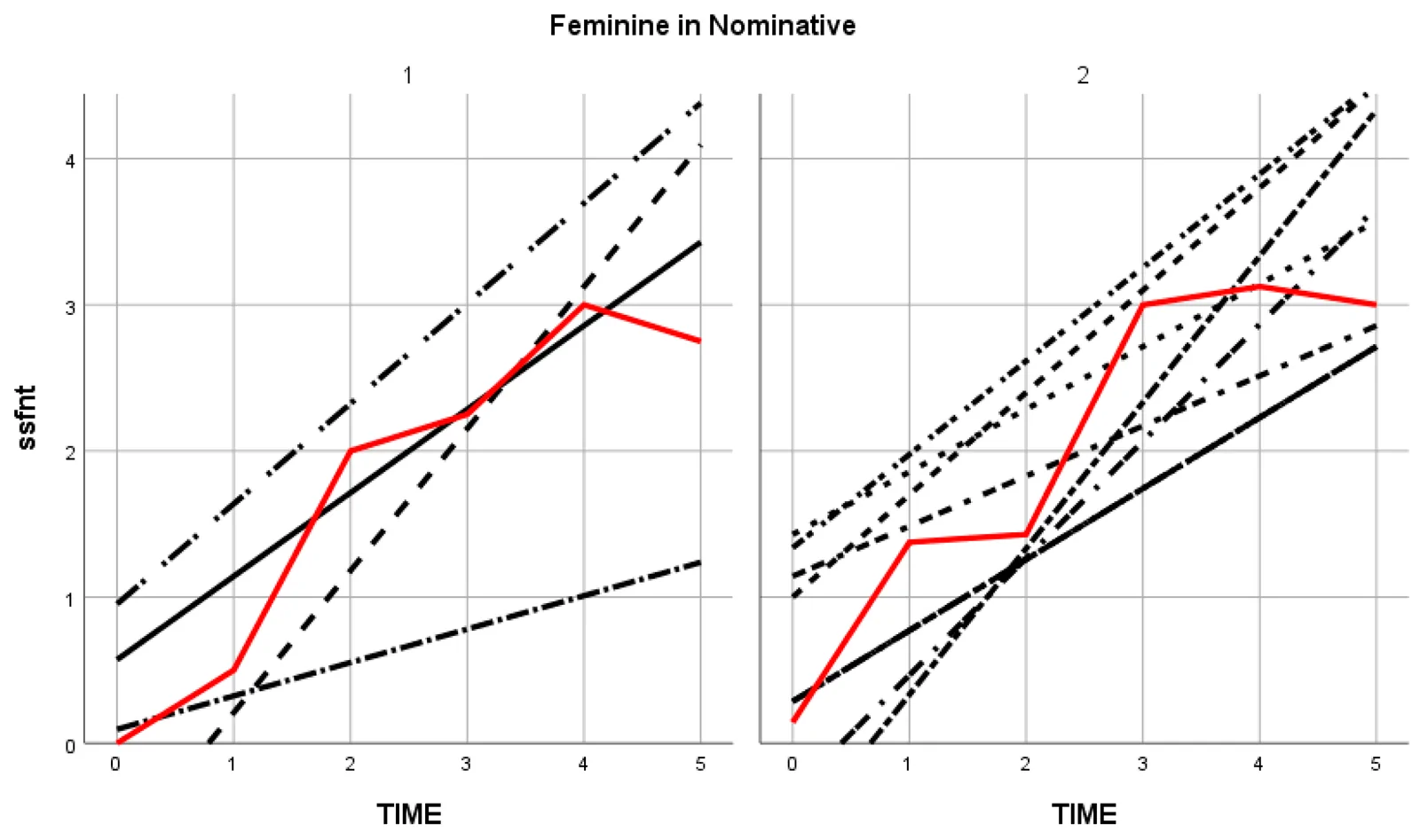
SSFNT group growth trajectories showing each group’s mean growth trajectory for the acquisition of the definite article used for the feminine gender in the nominative case. Graph 1 = CI group, Graph 2 = TH group.

**Figure 3:**
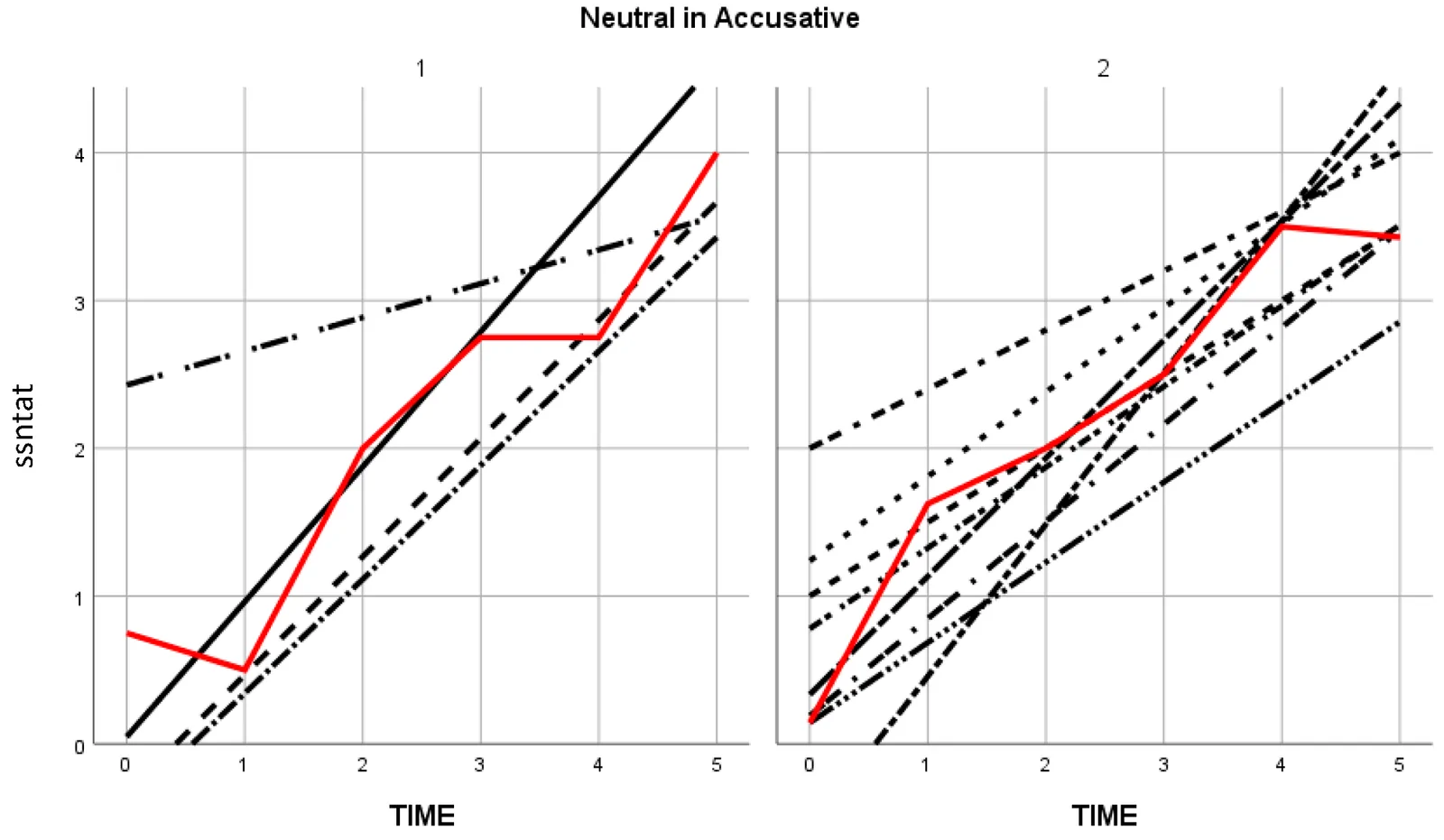
SSNtAT group growth trajectories showing each group’s mean growth trajectory for the acquisition of the definite article used for the neutral gender in the accusative case. Graph 1 = CI group, Graph 2 = TH group.

**Figure 4:**
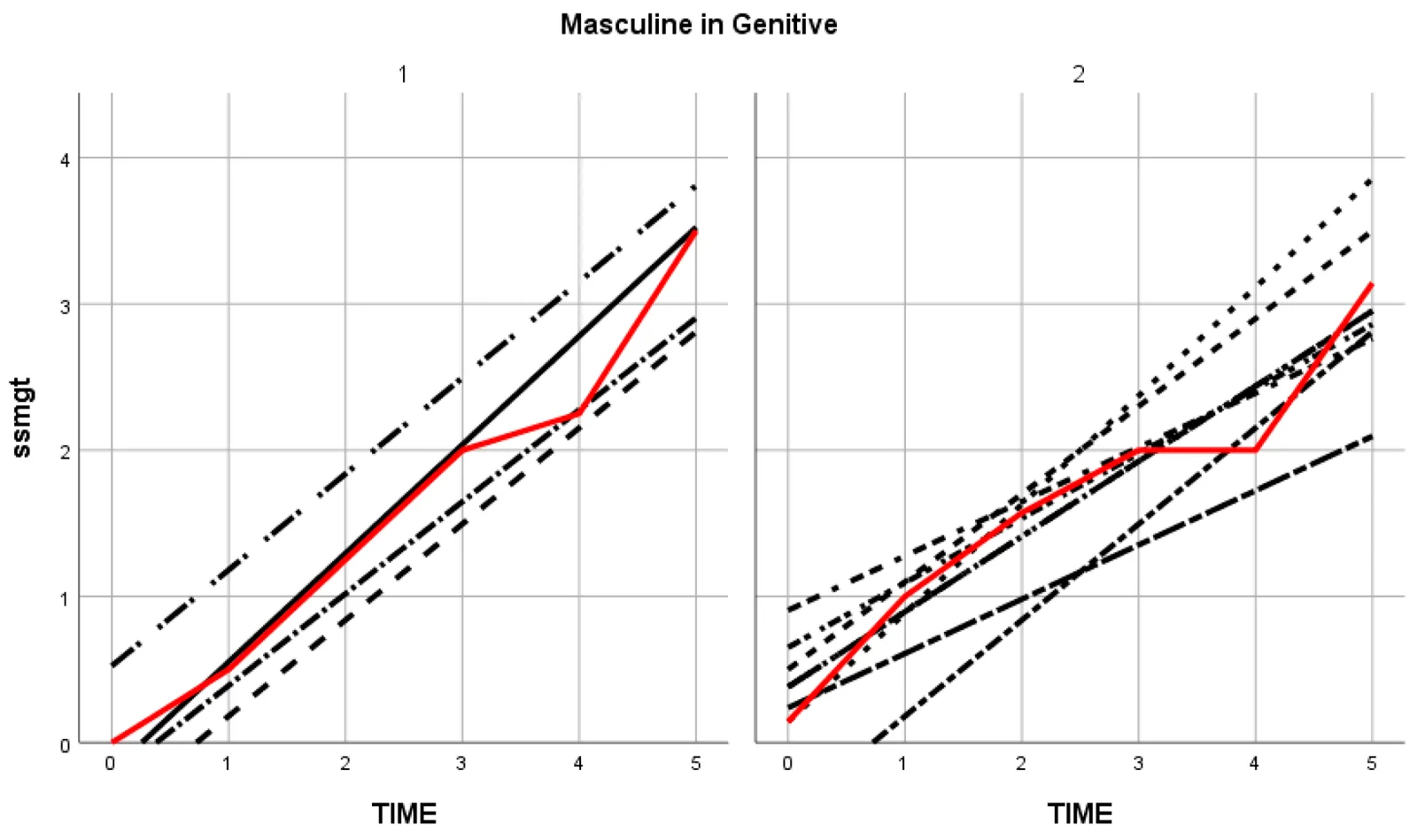
SSMGT group growth trajectories showing each group’s mean growth trajectory for the acquisition of the definite article used for the masculine gender in the genitive case. Graph 1 = CI group, Graph 2 = TH group.

**Figure 5:**
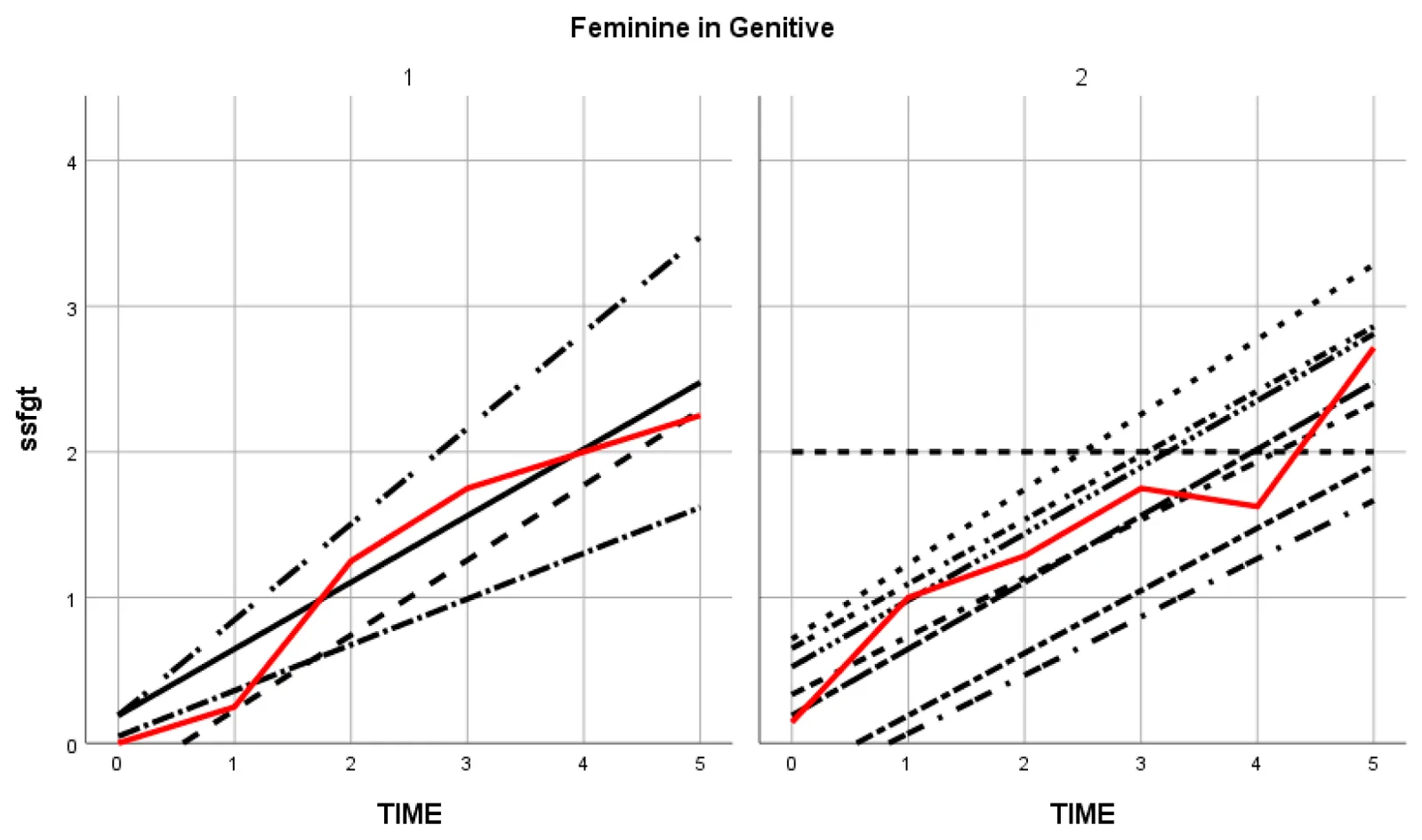
SSFGT group growth trajectories showing each group’s mean growth trajectory for the acquisition of the definite article used for the feminine gender in the genitive case. Graph 1 = CI group, Graph 2 = TH group.

**Figure 6:**
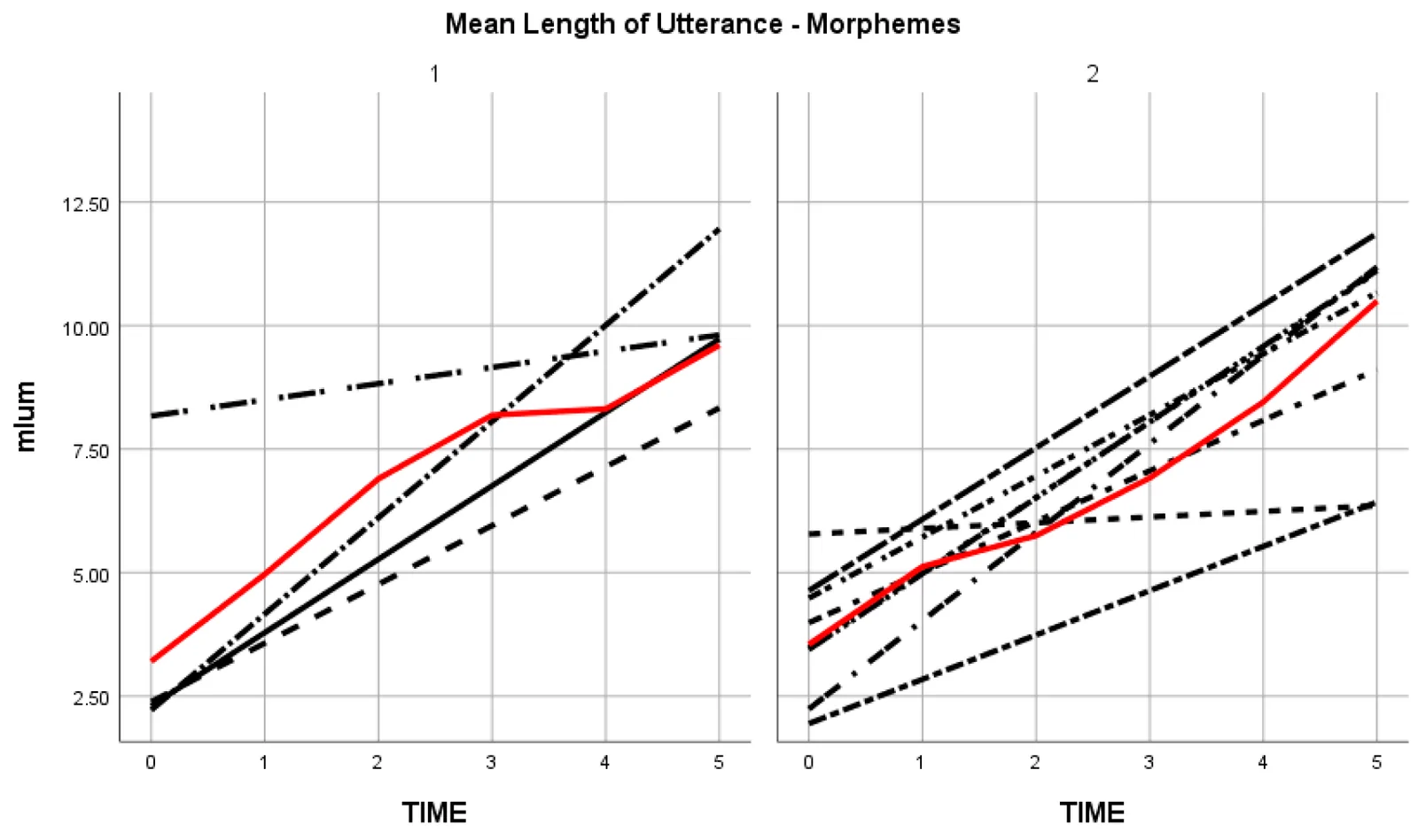
MLUm group growth trajectories showing each group’s mean growth trajectory for the Mean Length of Utterance in morphemes. Graph 1 = CI group, Graph 2 = TH group.

Figure 1, Figure 2, Figure 3, Figure 4, Figure 5 and Figure 6 present the descriptive group growth trajectory curves for the definite article forms for which the time effect remained significant after CITH was entered into the model. They are included to illustrate the shape of change over time for each cohort and are not intended to imply a statistically confirmed group difference.

In each figure, the solid lines represent the mean growth trajectories for the respective groups. For SSMNT (Figure 1), both cohorts showed an increasing trajectory between ages 2 years and 3 years; 6months, with the TH group showing descriptively higher accuracy for much of this period; the two groups’ means were close by age 4 years; 6 months. For SSFNT (Figure 2), both cohorts showed a generally increasing trajectory across the observation period, with some visual differences in pacing at individual age intervals.

Compared to other morphological structures, the masculine nominative (SSMNT) and feminine nominative (SSFNT) forms displayed descriptively steeper overall growth trajectories (Figure 1 and Figure 2). Both cohorts showed generally positive trajectories across the observation window, with some visual variation in pacing at individual intervals; as noted above, these differences did not reach statistical significance in the conditional model.

For SSNtAT (Figure 3), both cohorts showed an overall increase in accuracy across the observation period, with some visual alternation in relative pacing between groups at individual intervals. For SSNtAT (Figure 3), both cohorts showed an overall increase in accuracy across the observation period, with some visual alternation in relative pacing between groups at individual intervals. For SSMGT (Figure 4), individual growth trajectories clustered fairly closely around each group’s mean, with both cohorts showing generally steady development across the observation period. For SSFGT (Figure 5), both groups followed broadly similar average trajectories, progressing at a more gradual rate than observed for the nominative and accusative forms, consistent with the later emergence of genitive forms noted below.

Both groups began at comparable baseline MLUm levels and followed a broadly similar, generally linear increase across the observation period (Figure 6). As reported in Section 3.1.3, the conditional model did identify a statistically significant CITH main or interaction effect for MLUm. Descriptively, the CI cohort’s trajectory showed a brief plateau between ages 3 years; 6 months and 4 years that was not mirrored in the TH group, but given the sample size this pattern should be treated as illustrative rather than confirmatory.

## 4. Discussion

This longitudinal, exploratory case-series study examined patterns of emergence and use of the definite article across all genders, cases, and numbers, as well as growth in Mean Length of Utterance in morphemes in four early-implanted Greek-Cypriot-speaking children with CIs and eight TH peers. Given the sample size, the study is best characterized as hypothesis-generating rather than confirmatory, and the following three questions were treated as open research questions rather than directional hypotheses to be formally tested:

(1) Do all participants, irrespective of hearing state at birth, exhibit an overall increase in MLUm scores and correct occurrences of the definite article in all its forms and does participants’ age affect the direction in which their MLUm scores and number of correct definite article (DA) occurrences move?

(2) Is there a difference between the children with and without CIs in the early pattern of correct productions of the definite article in the nine forms examined?

(3) Is there a difference between children with and without CIs in their morphosyntactic level as measured using MLUm, MLUw and TTR?

**Do all participants, irrespective of hearing state at birth, exhibit an overall increase in MLUm scores and correct occurrences of the definite article in all its forms and does participants’ age affect the direction in which their MLUm scores and number of correct definite article (DA) occurrences move?**

Both cohorts showed a statistically significant, positive linear association between chronological age and both definite article accuracy and MLUm (Table 3 and Table 4). The fixed effect of time was highly significant across all twelve outcome measures (*p* < 0.0001), indicating that, descriptively, definite article uses and morphosyntactic complexity increased with age in both groups over the observed window. This is consistent with the possibility that early cochlear implantation supports continued growth in morphosyntactic skills, in line with earlier reports in other languages [2,8,9,10]. Because the CI group in this study consisted of only four children, this finding should be regarded as a preliminary observation rather than confirmation that early implantation is sufficient to fully offset the acoustic and linguistic demands of a highly inflected language; larger, adequately powered studies are needed to test this possibility directly.

While both cohorts’ trajectories moved in the same overall direction, the plots also suggested that periods of relatively faster or slower growth did not always occur at the same chronological ages in the two groups (Figure 1, Figure 2, Figure 3, Figure 4, Figure 5 and Figure 6). Importantly, the formal conditional models did identify significant group × time interactions for nine of the twelve definite article measures (Table 4), indicating that the rate of change over time systematically differed between groups for those forms. This stands in contrast to the descriptive “asynchrony” noted above and confirms that, for most DA forms, the developmental pace was conditional upon hearing status. One plausible account, consistent with critical-period models of auditory and language development [15,16,17,18,19,21,22], is that growth spurts in children with CIs may track hearing age (time since implantation) rather than chronological age, so that surges could appear at different chronological ages than in TH peers. We did not test this directly because hearing age was not entered as a separate predictor in the present models, a choice driven by the very small sample size and the resulting risk of overfitting a model with additional parameters; testing hearing age explicitly, ideally in a larger cohort, is an important direction for future research.

Despite this group-dependent variability in growth rates, both groups’ mean trajectories maintained the same overall upward direction, broadly consistent with catch-up growth patterns reported for children with CIs in other languages [64]. If replicated in larger samples, this pattern would suggest that the structural complexity of GC does not necessarily prevent children with CIs from following a developmental path broadly similar to that of their TH peers; at present, however, this remains a hypothesis rather than a demonstrated conclusion, and should not yet be used to guide specific clinical recommendations.

**Is there a difference between children with and without CIs in the early pattern of correct productions of the definite article in the nine forms examined?**

When examining the initial emergence of the nine grammatical forms of the Greek definite article, we found that the presence or absence of a significant group × time interaction differentiated the measures into two clusters. For most of the definite article forms—specifically SSMNT, SSFNT, SSNtNT, SSMAT, SSFAT, SSNtAT, SSMGT, SSFGT, and SSNtGT—the Kenward–Roger adjusted tests revealed statistically significant fixed interaction effects between time and hearing condition (*p* < 0.05; Table 4). These significant interactions indicate that the rate of change over the six measurement waves systematically differed depending on the child’s hearing group. Because the presence of a significant interaction establishes that the effect of time is conditional upon group membership, the main effects for these nine variables are not interpreted in isolation.

Within this interactive cluster, the Likelihood Ratio Tests evaluating individual-level random slopes for time revealed distinct patterns of individual variation. For SSMNT (*p* = 0.0001), SSFNT (*p* = 0.0001), SSFGT (*p*= 0.0001), and SSNtGT (*p* = 0.0001), the random slopes significantly improved model fit, indicating that individual children demonstrated unique baseline scores (random intercepts) as well as significantly different individual rates of progression over time (random slopes). Conversely, for SSNtNT (*p* = 0.096), SSMAT (*p* = 0.872), SSFAT (*p* = 0.285), SSNtAT (*p* = 0.12519), and SSMGT (*p* = 0.255), the random slope parameters were statistically non-significant, suggesting that while children varied significantly in their initial baseline skills, their individual growth trajectories over time progressed in a highly uniform and parallel manner around their respective group’s average slope.

Descriptively, both groups appeared to begin using the nominative forms of the definite article first, followed by the accusative forms and then the genitive forms. Genitive forms (SSMGT and SSFGT) emerged latest and showed the most uniform rate of growth across ages and initial status in both groups, consistent with patterns reported in other richly inflected languages [9]. This later emergence of the genitive forms relative to the nominative is consistent with patterns reported in other highly inflected languages, plausibly because genitive case marking carries a heavier semantic and thematic load [9]. In our data, both cohorts appeared to follow a broadly similar developmental sequence for these forms, which is compatible with, though not proof of, morphosyntactic development in children with CIs following a similar overall course to that of TH peers.

The later, comparatively steep rise in genitive forms observed in both groups around ages 4 years to 4 years; 6 months may reflect the additional cognitive and linguistic demands of this grammatical category, though this study cannot establish the underlying mechanism. If this pattern is confirmed in larger samples, one possible clinical implication worth testing is that temporary plateaus in grammatical production, including in children with CIs, need not necessarily be treated as signs of a deficit; we present this as a hypothesis for future research rather than as a clinical recommendation at this stage.

**Is there a difference between children with and without CIs in their morphosyntactic level as measured using MLUm, MLUw and TTR?**

A secondary profile was observed for Mean Length of Utterance in morphemes (MLUm), Mean Length of Utterance in words (MLUw), and Type–Token Ratio (TTR). For these three dependent variables, the fixed interaction effect between time and hearing condition was non-significant (*p* = 0.120, *p* = 0.142, and *p* = 0.119, respectively). This establishes that the rates of development across the six testing windows were statistically parallel between the TH and CI groups.

Descriptively, MLUm values for the two groups overlapped considerably across the observation window, with neither group showing a consistent or growing gap relative to the other; at the final time point (4 years; 6 months years), the TH group’s mean MLUm was descriptively somewhat higher, but this difference was not statistically significant. Taken together with the substantial between-child variability noted in Section 3.1.1 (within-subject variance of 3–26% versus up to 74% between-subject variance for several outcomes), these findings suggest that individual differences among children play a considerable role in morphosyntactic development in this sample, and that any conclusions about hearing status specifically should be treated as preliminary pending replication in a larger cohort. Because hearing age was not modeled directly and no formal equivalence test was conducted, we recommend for both to be incorporated in future adequately powered studies of Greek-Cypriot-speaking children with CIs.

### Limitations and Future Directions

The primary limitation of this study is the small sample size, which precludes robust generalization and limits the statistical power to detect smaller effects. As noted throughout, the analyses are exploratory and descriptive; all findings should be interpreted as hypothesis-generating rather than confirmatory. Future studies with larger samples are needed to replicate these findings, to formally test equivalence between groups, and to incorporate hearing age as a separate predictor alongside chronological age. Additionally, the lack of standardized language assessments for the GC dialect necessitated the use of parent report measures (CDI) for baseline inclusion; development and validation of dialect-specific standardized tools would strengthen future research.

## 5. Conclusions

Within the limits of this small sample, the findings suggest that early cochlear implantation supports continued morphosyntactic growth in Greek-Cypriot-speaking children, with chronological age driving overall development in both CI and TH groups. Significant group × time interactions for most definite article forms indicate that the rate of acquisition of specific grammatical morphemes differs between groups, while the parallel trajectories for MLUm, MLUw, and TTR suggest that general morphosyntactic complexity progresses similarly once early auditory access is established, albeit with a persistent baseline group difference. The substantial between-child variability underscores the importance of individual differences in language development. These preliminary observations require replication in larger, adequately powered studies before they can inform clinical practice or educational policy.

## Figures and Tables

**Table 1:** Hearing-impaired participants’ demographics.

Part.	Gender	CA Actual (A) Corrected (C)	HL (dB)	CI Impl. Age Right Ear	CI Impl. Age Left Ear	CI Activ./Hearing Aid (HA) Age	Hearing Aid Use Pre-Implantation	Bilateral (B) or Unilateral (U)	Etiology	Preterm (P) or Full-Term (F)
CI1	M	2;1(C)	95	1;1(A) 0;10(C)	0;9 (A) 0;6 (C)	R— 1;2(A) 0;11(C) L 0;10(A) 0;7(C)	Bilateral	B (sequential)	Prematurity and intake of antibiotics for an extended period of time	P—27 weeks
CI2	M	2;1	110(R) 105(L)	1;0	2;10	R—1;1 L—2;11	No HA	B (sequential)	Unknown	F
CI3	F	2;3	95(R) 100(L)	_	0;9	HA (R)—0;3 CI (L)—0;10	U (R) HA L ear	U (L)	Connexin	F
CI4	F	2;0	100	1;0	1;0	R + L—1;1	Bilateral	B (simultaneous)	Unknown	F

**Table 2:** Names for the nine forms of the Greek definite article obtained during the Structured Session.

	Masculine	Feminine	Neutral
Nominative	SSMNT	SSFNT	SSNtNT *
Accusative	SSMAT	SSFAT	SSNtAT
Genitive	SSMGT	SSFGT	SSNtGT

SS = Structured Session, M = masculine, F = feminine, Nt = neutral, N = nominative, A = accusative, G = genitive, T = total of occurrences with a max of occurrences = 2. * This variable was the only one with a total maximum of occurrences = 1.

**Table 3:** Significance values of time as a predictor of outcome variability.

		Time			
Outcome Variables	Estimate	Std. Error	Df	t-Value	Pr(>|t|)
**SSMNT**	0.586	0.066	10.912	8.829	0.0001 *
**SSFNT**	0.611	0.072	10.959	8.466	0.0001 *
**SSNtNT**	0.231	0.047	21.816	4.877	0.0001 *
**SSMAT**	0.530	0.075	11.139	6.994	0.0001 *
**SSFAT**	0.651	0.089	11.249	7.301	0.0001 *
**SSNtAT**	0.654	0.075	17.305	8.620	0.0001 *
**SSMGT**	0.574	0.042	44.287	13.603	<2 × 10^−16^ *
**SSFGT**	0.449	0.044	39.594	10.198	1.23 × 10^−12^ *
**SSNtGT**	0.321	0.061	12.685	5.233	0.0001 *
**MLUm**	1.267	0.138	9.834	9.184	0.0001 *
**MLUw**	0.353	0.067	10.389	5.211	0.0001 *
**TTR**	0.021	0.008	11.157	2.429	0.033 *

* Significance is reached when *p* < 0.05.

**Table 4:** Summary of *p*-values from nested multilevel model comparisons across 12 dependent variables.

Variables	Random Slope Variance (*p*)	Fixed Interaction: Time × CITH (*p*)	Fixed Main Effect: CITH (*p*)	Fixed Main Effect: Time (*p*)
SSMNT	0.000	0.011	0.888	<0.0001
SSFNT	0.000	0.016	0.343	<0.0001
SSNtNT	0.096	0.001	0.250	<0.0001
SSMAT	0.872	0.013	0.360	<0.0001
SSFAT	0.285	0.007	0.117	<0.0001
SSNtAT	0.125	0.002	0.625	<0.0001
SSMGT	0.256	0.005	0.618	<0.0001
SSFGT	0.000	0.021	0.809	<0.0001
SSNtGT	0.000	0.002	0.018	<0.0001
MLUm	0.000	0.121	0.031	<0.0001
MLUw	0.000	0.142	0.049	<0.0001
TTR	0.005	0.119	0.003	<0.0001

Note: Fixed effect evaluations utilize a Kenward–Roger F-test approximation; random slope evaluations utilize a Likelihood Ratio Test (χ^2^). Main effects for the first nine variables are included in the table for structural completeness but are not interpreted textually due to the presence of higher-order interactions.

## Data Availability

The data presented in this study are available on request from the corresponding author due to privacy.

## Abbreviations

The following abbreviations are used in this manuscript:

CI	Cochlear implants
TH	Typically hearing
MLUm	Mean Length of Utterance in morphemes
MLUw	Mean Length of Utterance in words
TTR	Type/Token Ratio
SSMNT	Structured Session Masculine Nominative Total
SSFNT	Structured Session Feminine Nominative Total
SSNNT	Structured Session Neuter Nominative Total
SSMAT	Structured Session Masculine Accusative Total
SSFAT	Structured Session Feminine Accusative Total
SSNAT	Structured Session Neuter Accusative Total
SSMGT	Structured Session Masculine Genitive Total
SSFGT	Structured Session Feminine Genitive Total
SSNGT	Structured Session Neuter Genitive Total

## Appendix A

### Appendix A.1. MLUm Coding Rules

1. Each morpheme gets one point;
2. In nouns there is a maximum of four points: one—noun (just saying the word), one—gender (M, F, N), one—number (Sn or Pl), and one—case (Nom, Acc, or GEN), e.g., the word κροκόδειλος will be coded as follows: **N:M:Sn:Nom;**
3. The same applies for adjectives, e.g., μεγάλος will be coded as follows: **Adj:M:Sn:Nom;** again there is a maximum of four points a child can get;
4. The definite articles (DAs) are scored in the same way as outlined above. The only difference is the coding will start with DA: ….;
5. The same applies for personal, possessive and indefinite pronouns (της, την, των, τους, μου, σου, άλλα, άλλους, κάποιοι κτλ.) The indefinite pronoun ‘κάτι’ is always undeclinated. Again, there is a maximum of four points, e.g., in “H αγελάδα μου */I agelada mu/*” *(my cow)*, the possessive pronoun μου */mu/ (my)* will be coded as follows: **PossPr:1st:Sn:GEN** (one—PossPr, IndPr, PersPr; one—person (first, second, third), one—number (Sn or Pl), and one—case (Nom, Acc, GEN);
6. For verbs there is again a maximum of four points: one—verb (V), one—person (first, second, third), one—number (Sn or Pl), and one—tense (present tense (PrsT), past tense (PT), future tense (FT), present subjunctive (PrsSubj), future tense (FT)), e.g., the verb παίζει will be coded as follows: **V:3rd:Sn:PrT;**
7. For each of the four aspects for the parts of speech outlined above, if the child gets wrong, they lose that point. The correct gender, number, case, or tense needs to be put in parenthesis before the incorrect form used by the child, e.g., “Να ο παπαγάλο */na o papagalo/” (there’s the parrot)* will be coded as follows: N:M:Sn:(Nom)Acc; in this case the child has put the noun in the wrong case. Therefore, this word receives three points instead of four;
8. If the child uses the wrong definite article (DA) and the ending of the word cannot determine the gender because it could be feminine or neutral then the gender is not considered correct in either of them (definite article or noun), e.g., O πεταλούδα */o petaluda/ (the butterfly)* will be coded as follows: DA:(F)M:Sn:Nom; N:(F)M:Sn:Nom;
9. If the DA and the ending of the word match the gender of the noun, but one of the two indicates a different case then the error is in the choice of case, as in the example above ‘Να ο παπαγάλο’. There is an obligatory context for the use of nominative case. The DA is in Nom but the ending of the noun indicates use of Acc. The error is therefore in the use of case;
10. Every other part of speech is appointed one point. These include adverbs, interrogative pronouns, and conjunctions;
11. If the child uses the wrong adverb, interrogative pronoun, or conjunction, do not deduct the point. The point is awarded for simply using that particular part of speech;
12. Stereotypes like Έπεσε */epese/*, μπάνιο */banio/*, ένα λεπτό */ena lepto/*, περίπατο */peripato/*, βόλτα */volta/*, και έννε */enne/* are awarded only one point;
13. The route phrase πούντο */punto/* is awarded one point if it is not declinated according to gender and number. It is awarded two points if it is declinated according to gender and number;
14. For single word utterances where the child does not demonstrate knowledge of gender, number and case, do not award points assuming the child knows the gender, number or case of the noun. For example, for the noun αλογο, the coder will award points for N (noun) and Sn (number). The coder will not award points for case as the noun is not preceded by a definite article indicating the case. For the noun τραπέζι, the coder will award points for N (noun) and Sn (number) only as the ending -ι of the noun could be either feminine or neutral phonemically. Case will not be granted one point as it is not evident due to the lack of the definite article;
15. Fillers, such as εμμμ, να να να, το το το, and ε…να…το…μα…, do not receive any points;
16. Animal sounds do not count;
17. Phrases such as γεια σου, γεια σας are not given any points;
18. If the child repeats what the examiner said without adding to the phrase or adapting it according to his/her intended utterance, it does not receive any points. It is coded as ‘repetition’ and not included in the final utterance total count;
19. If the child repeats a specific phrase within his/her utterance then only one instance of that phrase should be coded;
20. Incomplete utterances are coded as ‘incomplete’ and are not included in the final utterance total count;
21. For the phrase ‘δικό σου/μου/του’, etc., count as one possessive pronoun;
22. For ‘εν να’ count as one and code as participle (Partic);
23. For phrases like ‘να παίξει’, να is the subjunctive and the verb is in future simple tense. For phases like ‘να παίζει’, να is subjunctive and the verb tense is present subjunctive;
24. Verbs preceded by the participle ‘θα’ or ‘εννα’ are in the future tense;
25. The verb ‘είναι’ is awarded one point as this is the standard form for children. If it is declinated then it is awarded all the necessary points as with any other verb;
26. All words in the Cypriot dialect are counted correct and are awarded the necessary points as are words in Standard Modern Greek;
27. Any words in English are coded as ‘other’.

### Appendix A.2. MLUw Coding Rules

1. Count all the words in each utterance, add them up and divide by the total number of utterances uttered by the child;
2. Do not include words that are repeated meaninglessly within an utterance. All words count except for animal sounds;
3. Stereotypes receive one point;
4. Route phrases such as ‘γεια σου’ ‘κι άλλο’ count as one word and are therefore awarded one point;
5. The subjunctive and future participles *να* and *θα* do not count as separate words. Award points according to the verb following them and its declination.
